# Development and Validation of a PET/SPECT Radiopharmaceutical in Oncology

**DOI:** 10.1007/s11307-021-01645-6

**Published:** 2021-09-20

**Authors:** Federica Pisaneschi, Nerissa T. Viola

**Affiliations:** 1 Department of Cancer Systems Imaging, MD Anderson Cancer Center, Houston, TX 77030 USA; 2 Department of Oncology, Karmanos Cancer Institute, Wayne State University, Detroit, MI 48201 USA

**Keywords:** Radiopharmaceuticals, Radiotracers, PET, SPECT, Radiochemistry

## Abstract

In oncology, biomarker research aimed to provide insights on cancer biology via positron emission tomography (PET) and single photon emission tomography (SPECT) imaging has seen an incredible growth in the past two decades. Despite the increased number of publications on PET/SPECT radiopharmaceuticals, the field lacked standardization of in vitro and in vivo parameters necessary for the characterization of any radiotracer. Through the efforts of the World Molecular Imaging Society Education Committee, this white paper lays down validation studies that are essential to chemically and biologically characterize new radiopharmaceuticals derived from small molecules, peptides or proteins. Finally, a brief overview of the steps toward translation is also presented.

Herein, we discuss the following:

- Chemistry and radiochemistry metrics to establish the identity of the imaging agent.
- In vitro and in vivo studies to examine the radiotracer’s mechanism of action, which includes target specificity, pharmacokinetics and in vivo metabolism.

## Introduction

As per the World Health Organization definition, “Radiopharmaceuticals are unique medicinal formulations containing radioisotopes which are used in major clinical areas for diagnosis and/or therapy”. The most widely clinically used radiopharmaceutical, [^18^F]fluorodeoxyglucose ([^18^F]FDG), employed for the diagnosis and staging of cancer by Positron Emission Tomography (PET), accounts for the vast majority of the over 2 million PET-scans reported in 2020 in the USA. In the last two decades, a plethora of new radiopharmaceuticals have been reported in the preclinical literature, with some that were translated to early clinical trials and a small but significant number that were approved by the FDA for clinical use. The latest have been prostate-specific membrane antigen (PSMA) PET agents for prostate cancer, with ^68^ Ga-PSMA 11 granted approval to UCLA and UCSF on December 1, 2020 [1] and ^18^F-DCFPyL (Pylarify) approved in late May 2021 [2].

One of the challenges in maintaining consistency when reporting data about production and preclinical validation of new radiopharmaceuticals is the lack of guidelines or standards that need to be met in order to provide the community with uniform and reliable data. The present white paper will address some of these issues and lay down a list of key parameters, albeit not exhaustive, in an effort by the WMIS Education Committee to standardize reports on the development of new radiopharmaceuticals.

## Chemistry/Radiochemistry

Radiopharmaceuticals are diagnostic or therapeutic tools and are produced in a highly regulated environment, following protocols that are set with high standards of safety. As with many other drugs, the final deliverable must have a high degree of purity, but, unlike standard pharmaceuticals that can be produced in lots and stored, radiopharmaceuticals, especially the ones labeled with the short-lived isotopes, must be produced “fresh” before use. This feature lays down a series of needs that must be addressed during the development of a new radiopharmaceutical.

*A new radiopharmaceutical must be produced using a reliable and reproducible synthetic method* [3] *and the consensus nomenclature rules for radiopharmaceutical chemistry must be obeyed* [4–6]*.*

For ^11^C- and ideally for ^18^F-tracers, it is preferable for the synthesis to be automated [7]. Many laboratories prefer to perform hands-on ^18^F-radiochemistry on novel radiotracers and invest in automation only when the biological value of the compound is proven. However, this must be balanced with the fact that manual ^18^F-radiochemistry typically adds unnecessary time to the development of an ^18^F-tracer because the corresponding automated synthesis can require a re-design and re-development of the whole synthetic process, steps that can be avoided if the radiosynthesis is conceived directly in automation. In addition, synthetic modules often have a built-in system for purification and reformulation of the radiopharmaceutical which allow for optimization of the entire procedure from end-of-bombardment to final formulation. Morever, an automated synthesis minimizes risks of human error, adding to the reliability of the process and often allows production of higher doses, enabling more complex biological studies. With radiometals, where the labeling procedure is usually straightforward incubation method, hands-on syntheses are still very common, although small synthetic modules are available for preclinical development [8, 9] and routinely used for human studies [10–12]. High activity yields (non-decay corrected) are of course desirable, but for ^11^C- and ^18^F-chemistry, a 3–4% activity yield from end-of-bombardment (EOB) to formulation is acceptable for initial validation. Decay corrected radiochemical yields can be a useful indication of the efficiency of the labeling method, and are commonly used in radiochemistry methodology development. They, however, have less meaning in radiotracer development, and, when reported, should be alongside activity yields. The length of the synthetic process, from end-of-bombardment to formulation, and a confirmation of the % purity is also paramount prior to utilization in biological systems.

Any new reported radiotracer must be radiochemically and chemically characterized. Identity must be confirmed by high performance liquid chromatography (HPLC) via co-elution with the non-radioactive reference compound, with one, and preferably two different mobile phases. Since radio-HPLCs are usually equipped with a γ- and a UV detector, a UV-trace is usually presented as proof of identity. If the final compound is not UV-active throughout a multistep synthesis, the identity of the UV-active intermediate (e.g. the protected analogue of the final compound) must be reported [13]. Other types of detectors, such as fluorescence and refractive index detectors, are equally valid. Mass spectrometry (MS) via a HPLC–MS is also an acceptable alternative for the detection of small amounts of carrier observed in most radiotracer preparations, even at high molar activities [14]. The UV trace of the final compound should be reported as proof of chemical purity and ideally will show only background noise. Although an accurate analysis of chemical non-radioactive (solid) contaminants is not required for preclinical validation, quantification of UV-contaminants can be reported, under the assumption that all peaks have an identical extinction coefficient to the product of interest. If a major peak is shown in the UV-chromatogram, identification of the peak is desirable even in a preclinical setting. However, in the case where the tracer is used in human studies, the peak(s) absolutely must be identified. Assessment of the radiochemical purity via radio-HPLC as the ratio between the desired compound and the sum of all other radioactive peaks should be at least 95%.

Radiochemical yields and purities for proteins (e.g. antibodies) are most often determined using radio-instant thin layer chromatography (radio-iTLC), which identifies free isotope from the radiolabeled protein. The high pressure of HPLC systems, along with the use of organic solvents, can pejoratively affect the tertiary structure of antibodies. Certain conventional HPLC systems can be outfitted with size exclusion columns and aqueous solvents and employed in the quality control or separation of radiolabeled proteins. Alternatively, dedicated fast protein liquid chromatography (FPLC) systems are very convenient for quality control and separation of antibody aggregates or dimers that result from long term storage or high temperature incubations. In the absence of an automated liquid chromatography system, purification can also be performed using a size-exclusion column or a centrifugal spin column filter with molecular weight cut-offs ranging from 10 to 50 kDa with centrifugal speeds typically at ~ 3000–4000 rpm (400–1000 × g with a rotor radius of 4–6 cm). Unfortunately, there is no existing method to separate unlabeled from labeled protein.

The molar activity (MA) or specific activity (SA), expressed in SI units as either MBq/μmol to GBq/μmol or MBq/ μg to GBq/μg respectively, should be reported as a range. Although higher MAs often translate into superior performance of the radiopharmaceutical, it is difficult to predefine what range of MA is acceptable or not, as it typically depends on the biological systems that are targeted. Some systems are virtually unsaturable (e.g. hypoxia), so high MAs/SAs are not a requirement for good quality images. In receptor imaging, when the target protein is present in low concentrations and is easily saturated, radiolabeled antibodies typically provide good quality images with a SA in the order of MBq/mg, which is relatively low compared to small molecule agents. This stems from the antibodies’ high affinity for their targets, slower clearance from circulation and internalization potential, with consequent recycling of the target. Small molecules, on the other hand, need to be produced in very high MA, because they often have lower affinity, may compete with high concentrations of the physiological substrate of their target, and exhibit rapid clearance. Typically, a SA of 8–10 GBq/μg and a MA higher than 20–30 GBq/μmol for small molecules are considered acceptable. For radiolabeled proteins, a SA of > 74 MBq/mg is typical with as high as 740 MBq/mg reported. Lower SA values maybe be acceptable; however, this comes with the caveat that SA affects the direct quantification of receptor/antigen sites. As an example, antibodies with lower SAs and therefore more non-radiolabeled molecules, can saturate binding sites. This consequently leads to a decrease in signal. While higher established SAs are preferred, if the antigen of interest is shed in the blood, the tracer is likely sequestered by the circulating antigen. Another factor to consider is the “antigen sink”, wherein the antigen of interest (e.g. PD-L1) is found in non-tumor targets (e.g. bone marrow, spleen, liver, kidneys). In this case, lowering the SA/MA is beneficial as non-labeled proteins or carrier-added tracers will suppress non-specific tracer binding in antigen-expressing normal tissues while increasing uptake within the tumor [15–17]. Thus, a fine balance between SA and receptor/antigen density both in the organ/tumor of interest and the non-target tissues is needed for optimum imaging, detection and target monitoring. For both proteins and small molecules, high MA/SA yields a radiotracer that can be administered as a “microdose” as defined by the FDA (https://www.fda.gov/media/107641/download) and limits any concern about pharmacology effects of the radiotracer. This means that the injected mass is virtually non-existent, and the molecule itself only acts as a vector for the radioactivity and exerts no pharmaceutical effect nor elicit unwanted toxicity.

Site-specific conjugation of the chelator or prosthetic groups is paramount to the radiopharmaceutical’s design considerations. The moiety should be attached in regions where it does not hinder nor affect binding of the molecule to the receptor’s epitope or docking site. Another key component to characterize radiolabeled proteins is to determine the number of radiolabeling moieties (e.g. chelates), which is especially pertinent to radiometal-labeled antibodies/proteins. Determination of the number of chelators historically has been done through radiometric isotopic dilution assays [18]. Currently, the ratio of chelator:protein is characterized by mass spectrometry of the non-radiolabeled chelator-protein conjugate, which determines the number of attached molecules based on changes in mass between the parent and modified molecule [19].

Shelf stability of the final formulated compound (in saline or PBS with 10% ethanol or less) must be monitored via radio-HPLC with samples analyzed at different times, at least up to 4 h, at room temperature. Stability tests at 4 °C is also recommended. If radiolysis is observed, stabilizers,such as ascorbic or gentisic acid, can be added to the final formulation to attenuate this effect [20]. In vitro plasma stability, is an important information to add to the chemical characterization of the radiotracer and provides insight of its stability in vivo. This is obtained by incubation of the radiotracer with human and/or mouse plasma at 5, 30, and 60 min for short-lived radioisotopes, and at 24 h to as long as 144 h for longer-lived isotopes, depending on the isotope’s half-life.

## Examining the biology/mechanism of action

Although the concept of “biological validation” is potentially as broad as one can possibly imagine, a few minimal key but non-trivial experiments are required to demonstrate that the radiopharmaceutical visualizes the biological process or pathway for which it has been designed. The main goal of these biological validation experiments is to ensure that the radiopharmaceutical specifically interacts with the biological target and is not just retained in a non-specific fashion. In vivo stability, and in vivo biodistribution are also a few of the basic requirements for tracer characterization.

### *In vitro* studies and cross-validation

Although the present white paper is focused on radiopharmaceuticals developed for oncological applications, many concepts described herein are likewise applicable to tracers developed for other diseases. In oncology, the most common targets for radiopharmaceuticals are oncoproteins that are linked to key deregulated pathways. Evidence of target-mediated uptake must be shown in vitro via incubation of the tracer with cells expressing the target. Elevated radiotracer levels in these target-expressing cells confirms specificity versus low to negligibly bound tracer values in negative or low-expressing control cells. Target-positive cells can have a natural abundance of the target, such as ad-hoc tumor cells. Alternatively, cells can be engineered to express or downregulate the target, essentially creating isogenic models with similar genetic backgrounds. Normal tissue cells or cell with low expression of the target must be included as control. Expression has to be cross-validated by a different technique, the most common being western blots. Immunohistochemistry (IHC), bioluminescence imaging (BLI), flow cytometry or any technique able to independently confirm the expression level of the target adds value to the validation.

Additionally, target-mediated uptake must be confirmed with an intervention aimed at reducing the signal. This can include a variety of techniques, some more commonly used than others. Addition of an excess of the cold reference compound — at least 10 × , and up to 100 × , more than the radiotracer, aimed to competitively saturate the target and reduce the uptake of the radiopharmaceutical, the so-called “blocking experiment”, is perhaps the most common proof of target-mediated uptake of the drug, as opposite as non-specific uptake. Alternatively as discussed above, engineered knockdown cells or cells treated with drugs to modulate the target’s expression (i.e. inhibitors) can be employed. When possible, demonstrating differential tracer uptake in a panel of cell lines that have varying levels of target — usually high versus low — provides a thorough proof of target-mediated uptake of the radiopharmaceuticals. These cell lines either possess wild-type, knockdown or knock in target expression, or modulated via treatment with inhibitors using the drug vehicle as a control. Specificity of the tracer can also be confirmed by comparing uptake of a non-specific tracer. Low cellular uptake of a scrambled peptide or a non-specific mAb isotype validates specificity of the tracer.

Along these lines, peptide and antibody-based radiopharmaceuticals should be investigated for internalization kinetics upon binding to the target antigen. Tracer internalization is usually demonstrated as a function of time with incubation periods ranging from minutes to days. Information on the rate of tracer internalization renders meaningful insights on outcomes of radioligand therapy and dose selection as determined via image-guided PET or SPECT imaging.

For radiolabeled antibodies, the immunoreactivity or immunoreactive fraction (IF} is determined to establish what fraction of the radiolabeled mAb retains its ability to bind to its target antigen. The IF of a modified mAb is typically significantly less than 100% due to chelate/linker conjugation, radiosynthesis conditions, and damage from radiation during storage [21, 22]. During preclinical development, establishment of the IF of new tracers is a prerequisite. There is currently no consensus or defined acceptable range. Experts in the field deem acceptable immunoreactivity limits are > 70% if using live cells and > 80% if cell-free or immobilized antigens are utilized. Values below this range will likely affect targeted uptake of the agent as well as non-specific binding and clearance. There is no standard approach to determining IF. The most common protocol was developed by Lindmo et al*.* wherein the IF is linearly extrapolated to conditions that are under infinite antigen excess [23]. This method is deemed to derive the “true” IF versus the apparent IF value, determined under limited excess antigen conditions. However, the Lindmo protocol has drawbacks especially when antigen density on the cell surface is unknown or limited [24, 25].

The binding affinity, defined as the strength of association between the drug and its target ligand, is paramount when designing a mAb- (or small molecule)-based radiopharmaceutical where the radiolabeled compound is targeting a cell surface receptor, integrin, or other type of protein. Affinity is typically expressed as the equilibrium dissociation constant, K_D_, which is the ratio of how rapidly the drug dissociates (k_off_) to how fast it binds (k_on_). Thus, the smaller the K_D_, the higher the binding affinity. It is commonly thought that the highest affinity mAbs (10^−10^–10^−12^ M) are usually favored in drug development. However, in the case of tumor delivery, evidence suggests the mAbs do not distribute homogeneously throughout the tumor. They preferentially localize in the tumor periphery, within close proximity to blood vessels as a consequence of slow dissociation kinetics (k_off_ ~ 28 h) [26, 27]. This creates a binding site barrier, where mAb diffusion into the tumor core is hindered [28]. In contrast, low affinity mAbs (K_d_ > 10^−7^, k_off_ ~ 10 s) rapidly dissociate and move further into other regions of the tumor, but with the drawback of low tumor uptake and retention. A K_D_ “sweet spot” ranges between 10^−8^–10^−9^ M. In contrast, the tumor penetration of small molecule radiotracers is not as dependent on K_D_, but still remains a critical parameter to be established in development. Collectively, binding affinity dictates a significant role in tumor association and penetration of radiopharmaceuticals for imaging and therapy.

A key parameter in the development of a radiopharmaceutical is its binding potential (BP). It is a key measure of receptor occupancy derived from in vitro radioligand binding experiments. BP is calculated as the ratio of the total density of receptors in a tissue (B_max_) to affinity (K_D_). BP = Bmax KD

Of note, measuring in vivo receptor occupancy preclude receptors bound to endogenous ligands. For in vivo imaging studies wherein radiotracer concentrations are very low and occupying only a small portion of available receptors, the BP is indicative of bound versus free tracer concentration in a state of equilibrium [29].

### *In vivo *validation

In vivo metabolism is a very useful indication of how long the tracer remains intact in vivo as it accumulates within the tumor target prior to degradation. *S*tudies are usually performed by extracting the tracer and/or its metabolites at specific time points from tissues of interest and by analyzing the extract via radio-HPLC. A thorough analysis involves extraction and analysis of several tissues of interest (*i.e.* blood, liver, kidney, heart, tumor, even spleen). At minimum, metabolites from the blood and urine are reported. Typically the % intact compound in the organ is normalized to an “organ blank”, where the compound is added to an organ ex vivo and then homogenized, centrifuged, and the amount of compound that remains with the pellet is taken into account. An example of how this is done is described by Boswell et al., [30] where the in vivo stability of ^64^Cu-chelates was determined. The % authentic intact ^64^Cu-ligand complex = %P x %C x [1 + (% Pellet/% Super)] x % E, where % P = purity of injectate as determined by radio-iTLC; %C = ^64^Cu chelator complex determined by integration of the size-exclusion HPLC chromatogram; % Pellet/% Super = the ratio of the radioactivity in the pellet and supernatant of the organ blank; and % E = the extraction efficiency of the harvested organ after injection of ^64^Cu-labeled chelate. A figure should report the HPLCs of the intact standard staggered with representative HPLCs of the organs analyzed. Analysis can be performed at times ranging from 5 min to 24 h post-injection or longer, depending on the PK of the tracer. In addition, % intact compound at any time point should be reported with standard deviation for a minimum of three animals [31]. In general, an in vivo metabolism study informs on the viability of the tracer to accumulate in the tumor while remaining intact within a certain timeframe. If the tracer degrades rapidly prior to reaching the target, poor contrast is potentially obtained stemming from non-target tissue binding of radiometabolites.

Ex vivo biodistribution is the standard for characterizing the pharmacokinetic (PK) properties of the radiotracer. However, when and if possible, PET or SPECT imaging based biodistribution is equally accurate and sometimes preferable. This approach requires fewer animals and maintains the context of size and local environment which is critical for detection. Dynamic imaging scans, particularly with small molecule tracers, are also useful for in vivo PK analysis. They should be reported as Time Activity Curves (TACs) and expressed as %ID/cc.

Each radiopharmaceutical is designed to target a specific biological phenomenon. Regardless, in order to exert its role, a radiopharmaceutical requires a mechanism of retention or delivery to the target tissue. Mechanisms of delivery or retention can either be protein/ligand binding, [32] metabolic trapping as is the case for [^18^F]FDG [33] or [^18^F]FAZA [34], an in situ secondary reaction such as phosphorylation, or, simply, a shift in polarity [35]. These methods of radiotracer uptake should be identified, and, when possible experimentally proven.

Proof of target-mediated uptake must be demonstrated in vivo. Tumor bearing mice must be injected with the radiopharmaceutical, and imaging must be taken at different time points. With mAb-based tracers, the mAb must be cross-reactive to the target antigen. Fully human or humanized mAbs will require testing in established human xenografts or patient-derived tumors with overexpressed targets using immunocompromised hosts. When possible and if cross-reactivity of the tracer to the target is not an issue, employment of syngeneic tumors in animal models with an intact immune system will provide a better understanding of the role played by the immune system on the tumor uptake of the radiotracer [36]. Engineered mouse models, such as transgenic mouse models or knock-out models, are a very valuable addition to the validation of a new radiopharmaceutical.

For diagnostic agents, it is paramount to identify the timepoint that provides the best signal-to-noise ratio. For short-lived isotopes such as fluorine-18, dynamic imaging must be taken for at least one hour, followed by static images. Time points vary depending on the half-life of the agent: for short-lived isotopes, static scans can be recorded up to 3 h post injection. For mAb radiotracers bearing longer lived isotopes (e.g. copper-64, zirconium-89), imaging as early as 1–4 h p.i. has been reported, followed by acquisitions at 24 through 144 h p.i. It is worth nothing that imaging of anesthetized animals for prolonged periods of time should be done with caution since certain anesthetics can alter biological processes, which can affect tracer distribution. This can be attenuated by acquiring dynamic scans for at least 10 min to evaluate the initial biodistribution of the tracer, then statically at the 1 h or at later timepoints.

For preclinical validation, quantification of PET imaging is usually reported as % injected dose per cubic centimeter (% ID/cc). Standardized uptake values (SUVs) are also used in the preclinical field and are the most commonly used metric in the clinical field. Quantification of the agent accumulation as tumor-to-muscle or, for orthotopic tumors, tumor to the corresponding healthy tissue is important metric for quantification of contrast. Similar to the in vitro experiment described above, target-mediated uptake can be proven by using tumors with different expression of the target, or the target can be modulated pharmacologically, by means of inhibitors or pharmacological doses of the corresponding non-radioactive compound.

When cancer metabolic pathways are targeted, it is important to clarify what enzymatic or signaling pathway is responsible for the tracer accumulation in the tumor cells. When the tracer is an analogue of an endogenous metabolite, often a direct readout of the enzymes responsible for the tracer’s entry into the tumor cells (transporters) is provided. Notable examples are [^18^F]FDG and [^18^F]fluoroglutamine ([^18^F]FGln), which are transported inside the tumor cells by mainly GLUT1/3 and ASCT2 [37, 38], respectively, and retained intracellularly shortly after. In this case, results of a metabolite analysis can strengthen our understanding of the relevant applications the tracer can be used for. [^18^F]FGln for instance, can be employed to assess the overexpression of ASCT2, that might or might not translate into overactivation of glutaminolysis. However, as an example, using [^18^F]FGln as direct pharmacodynamic marker of glutaminase inhibitors can be complicated, because glutaminase is downstream the “trapping” point for the PET tracer. It can, however, be correlated to glutaminase inhibition, if upregulation of the transporters is occurring in that context [39]. Once an initial validation is performed, numerous applications of a new radiopharmaceutical can be investigated, and the list can be endless. More mouse models, such as orthotopic tumors, syngeneic mouse models, or patient-derived xenografts, can be employed, together with various types of interventions.

Biological applications span from understanding the pharmacokinetics/biodistribution of a drug via molecular imaging by using a radiolabeled version of the drug [40], or understanding the pharmacodynamics of a drug by using an imaging agent that reports on a secondary mechanism [19]. Imaging can also be extremely useful in understanding early response to therapy both as positive predictor or negative predictor [34, 41–45]. Finally, most of the validations reported herein are applicable to the development of radiopharmaceutical for systemic radiotherapy, that however, requires further robust and controlled tests to assess efficacy.

## Clinical translation

If a preclinical validation is successful, an imaging agent can become a clinical candidate and the process of clinical translation can begin. It is beyond the scope of this white paper to provide a thorough outline of all the steps for successfully translating a novel radiotracer into first-in-human studies, which has been reviewed elsewhere [46]. Below is a brief overview on the general steps towards translation.

It is extremely important for the investigator to understand the process for bringing a candidate radiotracer from bench-to-bedside at their institution. This usually includes the development of the radiopharmaceutical in GMP conditions, familiarity with institutional rules for intellectual property coverage, execution of Material Transfer Agreements (if needed), Institutional Review Board (IRB) approval, outsourcing of the synthesis, finding the right funding to cover the costs to obtain Phase 0/1 investigational new drug (IND) approval and last but not the least, clinical investigators interested in leading a potential clinical trial. A treating physician, on board from an early stage, will help to define preclinical pilot experiments that will be important for the writing of the first clinical protocol. A biostatistician is also key to identifying the sample size or number of patients to recruit.

## Conclusions

Although translation to human is usually the goal, radiopharmaceuticals can be developed for a variety of reasons that span from probing molecular mechanisms in mouse models, to veterinarian application. Regardless of the final objective, a strong synthetic methodology and a very careful, rigorous, mechanistic-based preclinical validation is instrumental for the success of any new compound and its adoption by the broader scientific community.
